# Editorial: Global infectious disease surveillance technologies and data sharing protocols

**DOI:** 10.3389/fpubh.2025.1676987

**Published:** 2025-08-29

**Authors:** Jie Huang, Zhiyan Ding, Jiaying Li

**Affiliations:** ^1^School of Public Health and Emergency Management, Southern University of Science and Technology, Shenzhen, China; ^2^International Curriculum Center of RDFZ, Beijing, China; ^3^School of Civil Engineering, University of Sydney, Sydney, NSW, Australia

**Keywords:** global health, infectious disease, surveillance, data sharing, pandemic

In this Research Topic titled “*Global Infectious Disease Surveillance Technologies and Data Sharing Protocols*”, we issued a call for papers “*related to the research, practice and architectural design of surveillance technologies and data sharing protocols that empower infectious disease prevention and preparedness at a global scale*”. We specifically encouraged submissions showcasing innovative ideas and discoveries in wastewater-based surveillance, innovative contactless technologies that could be deployed in public transportation vehicles, especially those across national and regional borders. At the time of the first SARS outbreak in the beginning of this century, the concept of wastewater-based surveillance was unimaginable. Today, it has become a reality, presenting a promising component of an integrated global aircraft-based genomic surveillance network (1). We also welcomed technical contributions employing artificial intelligence (AI) and blockchain technologies to enable real-time, transparent global data sharing. As articulated in our initial call: “*Once the global pandemic situation could be monitored and checked on anyone's smart-phone, like those for weather and air pollution, the lofty ideology of global pandemic prevention will be realized, from bottom up*”.

This Research Topic accepted a total of nine articles, with four primarily focused on sampling and experimental methodologies (i.e. “**wet**” studies), while four focused on statistical and computational modeling (i.e. “**dry**” studies). In December 2022, at the moment during China's transition in COVID-19 control strategies, we published a commentary titled “*The World Needs a ‘Pandamic' Solution for a Pandemic Problem*” (2). There, we introduced the term “pandamic” (pan-da-mic). There, “da” refers to data applications widely used and needed to fight against and prevent pandemics, while “mic” means microbiology and, in particular, various omics technologies. Therefore, the concept of “pandamic” stresses the essential convergence of biotechnology (wet) and information technology (dry) in modern surveillance frameworks. A particularly noteworthy contribution in this Research Topic is the review by Lipsitch et al., titled “*Infectious Disease Surveillance Needs for the United States: Lessons from COVID-19*”. The authors presented a comprehensive roadmap for improving national and global infectious disease surveillance systems. Drawing on insights from the COVID-19 pandemic, the article identified critical data types and infrastructure elements necessary to support real-time decision-making. The authors emphasized the integration of diverse data streams, including mobility patterns, internet search trends, clinical diagnostics, and wastewater signals, into purpose-built, responsive systems. Importantly, the article highlighted the importance of equitable and locally adaptive systems, capable of informing interventions not only during acute crises but also for ongoing public health challenges.

Three of the wet studies feature the term “wastewater” in their titles, reflecting the growing importance of wastewater-based surveillance in the global infectious disease monitoring landscape. Jones et al. investigated the feasibility of using *wastewater from passenger ships* as a surveillance tool for viral pathogens crossing maritime borders. Their study demonstrated successful detection of SARS-CoV-2 and norovirus in blackwater collected from short-haul ferries operating between the United Kingdom and Ireland. These findings validated the potential of maritime wastewater-based surveillance for tracking pathogen transmission across international boundaries, offering an important monitoring tool in the context of international travel. Maida et al. presented *urban wastewater surveillance in Sicily* during the 2022/2023 influenza season. The temporal trends of influenza viral RNA in wastewater were found to mirror clinical case trends, indicating the potential of wastewater-based surveillance as a non-invasive and cost-effective complement to traditional influenza surveillance in urban European settings. Dinssa et al. conducted a longitudinal study of *SARS-CoV-2 in Ethiopian wastewater* throughout 2023. They found a high positivity rate in untreated wastewater samples and a strong correlation between viral RNA levels and COVID-19 case trends. Their work underscored the capability of wastewater-based surveillance in low-resource settings, where limited access to clinical diagnostics may lead to underestimation of infection prevalence. This work provided compelling evidence that wastewater-based surveillance can fill critical surveillance gaps in resource-limited contexts. The fourth study from Dama et al. described the implementation of an integrated specimen reference system in Burkina Faso. This system employed existing courier networks to transport human biological specimens for priority diseases including COVID-19 from district-level clinics to reference laboratories in Burkina Faso. This innovative system achieved >99% on-time delivery with preserved sample integrity, proving that scalable, cost-effective logistical infrastructure can significantly enhance disease surveillance outcomes, especially for time-sensitive or high-risk conditions like the COVID-19 pandemic. Together, these four studies exemplify diverse and pragmatic approaches to enhance the front-line data collection for infectious disease surveillance, spanning novel applications of wastewater-based surveillance to innovations in biospecimen logistics.

All four dry studies include “model(s)” in their titles and collectively reflect a broad spectrum of modeling strategies and regional applications. Bowie and Friston assessed the predictive validity of *a dynamic causal model (DCM)* for long-term outcomes of the COVID-19 pandemic. While DCM captured several key pandemic dynamics, it tended to overestimate deaths and hospitalization rates due to fixed assumptions about virulence persistence. Their work offered a critical reflection on modeling assumptions and proposed more adaptive model frameworks that incorporate evolving population immunity. Hou applied time-series and machine learning methods to examine the epidemiology of *hemorrhagic fever with renal syndrome (HFRS)* in relation to environmental drivers. By integrating meteorological and air pollutant data using distributed lag non-linear models and support vector machines, the study provided a refined seasonal risk framework for HFRS outbreaks, highlighting the role of air quality as a significant predictor of disease outbreaks. Zheng et al. evaluated *ARIMAX models to predict influenza incidence* in Fuzhou, China, incorporating air pollutants and meteorological indicators. They found that PM_10_ was a particularly strong predictor and demonstrated that the inclusion of environmental indicators improved model accuracy. These findings provided practical implications for real-time influenza forecasting and public health early warning systems. Vijayalakshmi et al. developed *an optimal control framework for dengue transmission* using fractional-order differential equations based on the Atangana-Baleanu Caputo (ABC) calculus. Their mathematical model accounted for both symptomatic and asymptomatic infections and demonstrated that immune boosting and clinical treatment strategies could significantly reduce disease burden when integrated into control policies. Collectively, these four modeling papers presented the richness and diversity of analytic approaches that can support infectious disease prediction, environmental risk assessment, and intervention optimization across varied geographic and epidemiological contexts.

The COVID-19 pandemic, once a defining global crisis, now feels like a distant memory. Yet today, its urgency has largely receded from public consciousness and institutional agendas. As Darwin's theory of evolution suggests, humans are remarkably adaptive. But adaptation should not become synonymous with complacency. This moment calls for difficult, but necessary questions: Has anything fundamentally changed in the academic, operational, or policy landscape of global public health? If a COVID-like pandemic was to emerge tomorrow, would policymakers and societies respond more wisely, more swiftly, and more effectively? In China, as of July 2025, the infectious disease currently making headlines is the Chikungunya virus, transmitted by mosquitoes (3). In response, public health authorities have encouraged the public to drain stagnant water and apply insect repellent. These measures, while useful, have remained largely unchanged for over a century. Such public health intervention should reflect the leap in infrastructure, technology, or governance that reflects the lessons of COVID-19.

The echoes of “*I have a dream*” from Martin Luther King Jr. and “*we choose to go to the moon*” from President Kennedy continue to inspire visionary thinking. In the realm of global infectious disease surveillance, what are the equivalent aspirations? Do we have a unifying “dream” or a collective “moonshot” in this space? Or are we still navigating a fragmented landscape of national agendas and disconnected efforts? Public health is classically defined as “*the science and art of preventing disease, prolonging life, and promoting health through the organized efforts and informed choices of society*” (4). While biology and medicine anchor the scientific foundation, the “art” lies in policy, culture, communication, and the complexities of human behavior. From this perspective, public health is therefore inherently interdisciplinary, but this very breadth also risks diffusion of focus and a lack of accountability. Without concrete systems and enforceable structures, the noble ideals of public health remain vulnerable to drift. In this context, the World Health Organization (WHO) should evolve from a reactive body to a proactive global leader. It should articulate a clear, realistic, and actionable strategy for global infectious disease surveillance that is able to propel nations into coordinated efforts. Much like the International Olympic Committee (IOC), which established universal anti-doping protocols and inspired a shared framework for athletic integrity, WHO should provide both the inspiration and the infrastructure to coordinate global health preparedness. It should not only be the moral authority but also the architect of scalable solutions, setting enforceable global standards and guiding strategic investments to ensure no country is left behind.

In our original call for papers, we referenced the global anti-doping protocol as an instructive model: “*An exemplary is the protocol of world doping control, where all nations are obligated by the International Olympics Committee (IOC) to be sampled at any time by a WADA accredited laboratory*”. We further developed this idea in a recent Viewpoint article, inspired by a simple yet striking observation that the headquarters of the World Anti-Doping Agency (WADA) and the International Civil Aviation Organization (ICAO) sit just 30 meters apart in Montreal (5). Though they govern vastly different domains in sports and aviation, respectively, these two organizations succeed through international cooperation, cross-border enforcement, and standardized protocols. We proposed that ICAO could adopt a system similar to WADA, integrating infectious disease surveillance into international air travel. If designed and implemented with scientific rigor, equity, and transparency, such a system could serve as the foundational architecture for real-time, scalable global infectious disease surveillance. This proposal is both concrete and feasible, and represents a meaningful step toward a coordinated, adaptive, and enforceable global response infrastructure.

## Concluding remarks

The nine papers in this Research Topic collectively demonstrate the global diversity, creativity, and commitment in advancing infectious disease surveillance and preparedness. From ferry ports in the UK to wastewater plants in Ethiopia, from dengue transmission modeling in India to influenza forecasting in China, these studies reinforce the critical need for both robust frontline data collection and sophisticated analytic capabilities. Together, they reaffirm the critical importance of interdisciplinary collaboration across epidemiology, data science, and technology. As the COVID-19 pandemic has shown, a real-time, transparent, and decentralized surveillance infrastructure is no longer aspirational but a necessity. We encourage the global public health community to continue pushing the boundaries of innovation at this intersection of technology, data science, and epidemiology, ensuring that scientific insights translate into operational readiness. We hope this Research Topic serves as both a reflection and an inspiration to harmonize science, policy, and technology in the service of global health security.

While ideals can inspire, only tangible frameworks and enforceable standards can drive meaningful change. This distinction, between dreams and actionable solutions, lies at the heart of this Research Topic. Without structures that hold governments and institutions accountable, without interoperable systems that support timely data sharing, and without enforceable global agreements that transcend national interests, even the most visionary declarations risk becoming symbolic rather than substantive. We call on researchers, policymakers, and global institutions to move from rhetoric to rigor, from ambition to architecture.
